# Supplementary material for: Identification of photoperiod-induced specific miRNAs in the adrenal glands of Sunite sheep (Ovis aries)
Source: Front Vet Sci. 2022 Jul 22;9:888207. doi: 10.3389/fvets.2022.888207 (PMC9354845; doi:10.3389/fvets.2022.888207)
Supplement: Supplementary file 1 [file Data_Sheet_1.ZIP › Supplementary file/miRDeep2 file.zip!/miRDeep2 file/Know_Structure/SPLP42A2/oar-mir-133.pdf]

miRBase precursor : oar-mir-133
Total read count : 686
oar-miR-133 read count : 668
remaining reads : 18

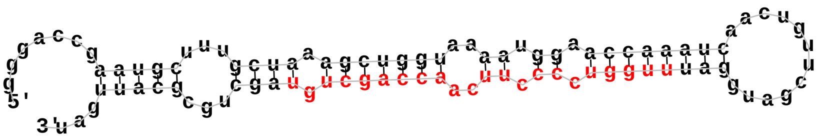

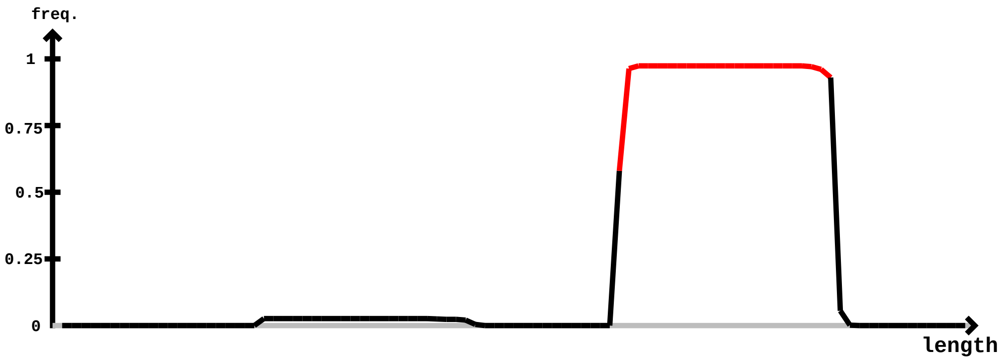

oar-miR-133

```
5'- gggaccgaaugcuuugcuaaagcugguaaaauggaaccaaaucaacuguucgauggauuugguccccuucaaccagcuguagcugcgcauugau   -3'    exp
    .......(((((...((((.(((((((..((.((.((((((((............)))))))).)).))..))))))).))))...)))))...     reads      mm         sample
    ....................agcugguaaaauggaacU........................................................     1          1          seq
    ....................agcugguaaaauggaacca.......................................................     1          0          seq
    ....................agcugguaaaauggaaccaaa.....................................................     2          0          seq
    ....................agcugguaaaauggaaccaaau....................................................     11         0          seq
    ....................agcugguaaaauggaaccaaauc...................................................     3          0          seq
    .........................................................uuugguccccuucaaccagc.................     2          0          seq
    .........................................................uuugguccccuucaaccagcu................     5          0          seq
    .........................................................uuugguccccuucaaccagcug...............     15         0          seq
    .........................................................uuugguccccuucaaccagcuA...............     3          1          seq
    .........................................................uuugguccccuucaaccagcugu..............     366        0          seq
    .........................................................uuugguccccuucaaccagcugua.............     7          0          seq
    ..........................................................uugguccccuucaaccagcu................     2          0          seq
    ..........................................................uugguccccuucaaccagcuA...............     1          1          seq
    ..........................................................uugguccccuucaaccagcug...............     2          0          seq
    ..........................................................uugguccccuucaaccagcugu..............     229        0          seq
    ..........................................................uugguccccuucaaccagcugua.............     28         0          seq
    ..........................................................uugguccccuucaaccagcuguag...........     1          0          seq
    ...........................................................ugguccccuucaaccagcugu..............     5          0          seq
    ...........................................................ugguccccuucaaccagcugua.............     2          0          seq
```
